# Unexpected bilateral pedicle stress fractures of the lumbar spine

**DOI:** 10.1016/j.radcr.2020.12.037

**Published:** 2020-12-30

**Authors:** Ismail Tahir, Muhammad Umar Islam

**Affiliations:** a University College Cork School of Medicine, College Rd, University College, Cork, Ireland,; b Fraser Health, Department of Radiology, Abbotsford, British Columbia, Canada

**Keywords:** Spondylosis, Pedicle, Stress fracture, Lumbar spine

## Abstract

Bilateral fracture of the lumbar pedicles is a rare phenomenon that may arise secondary to underlying risk factors. Our case demonstrates bilateral pedicle stress fractures arising in an otherwise healthy 41-year-old male. After failing to respond to conservative measures, he was managed with bilateral pedicle screws. Our patient did not report any incidence of trauma, but he did engage in amateur weightlifting – we suspect that this was the cause of his fractures.

## Introduction

**Fig. 1:**
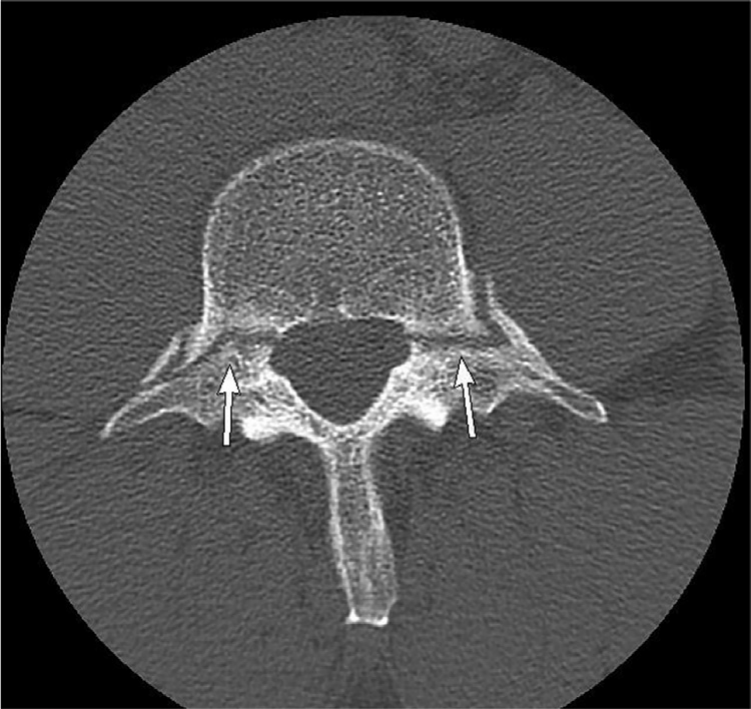
Axial CT showing bilateral transverse fracture of L4 pedicle.

**Fig. 2:**
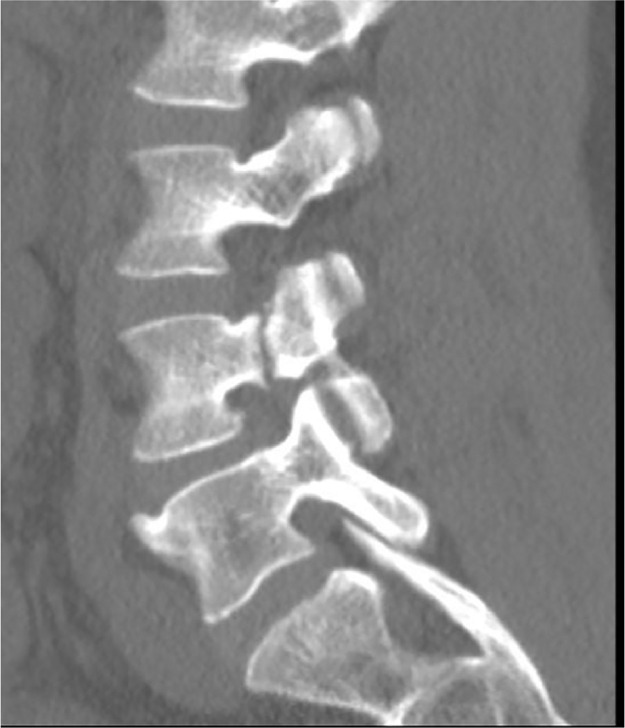
Sagittal CT image showing cranio-caudal extent of the fracture.

**Fig. 3:**
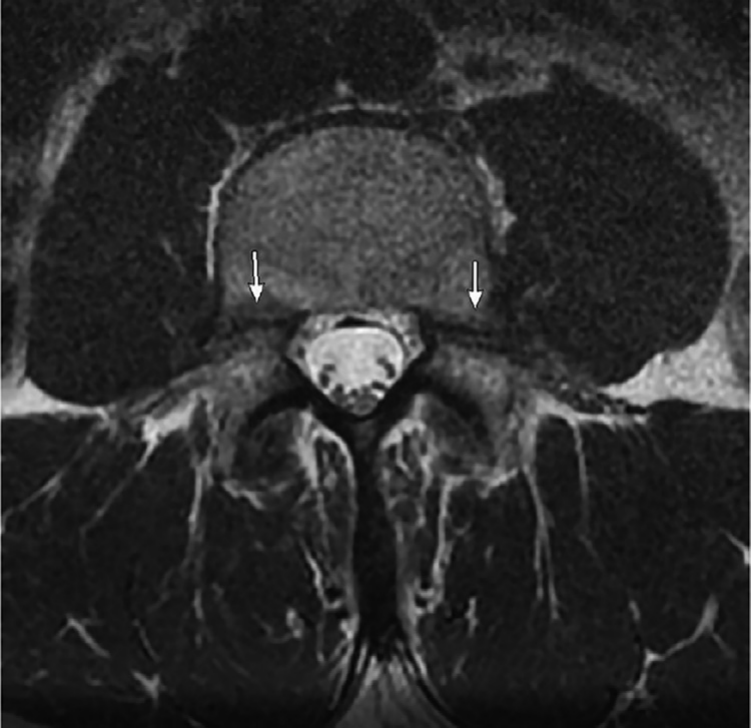
Axial T2-weighted MRI of L4 depicting bilateral pedicle fractures (straight arrow). No appreciable marrow edema.

**Fig. 4:**
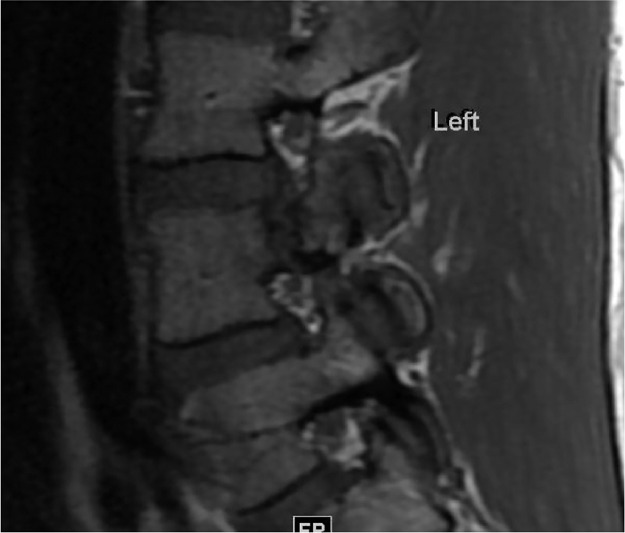
Sagittal T1 weighted MRI showing left pedicle fracture L4. Again, no marrow fat signal loss to suggest bone edema.

**Fig. 5:**
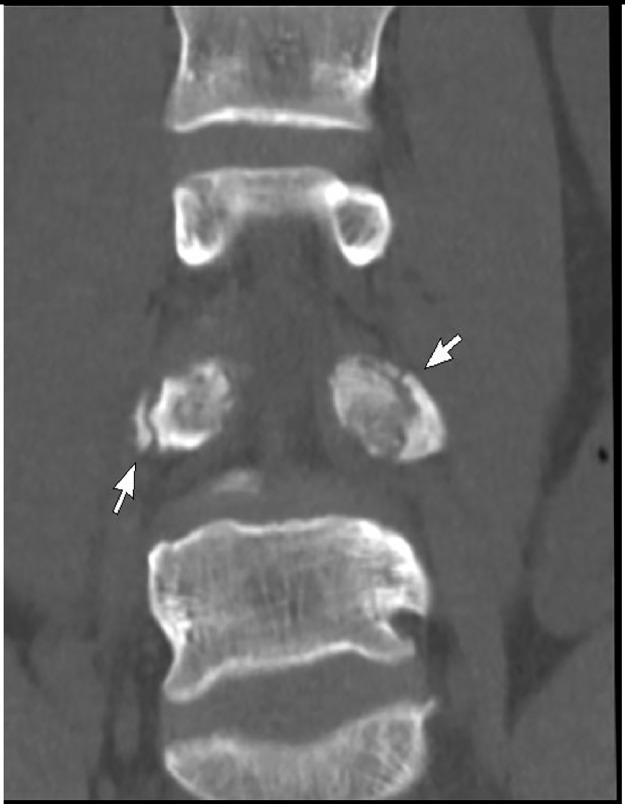
Coronal CT reformats showing bilateral fractures.

**Fig. 6:**
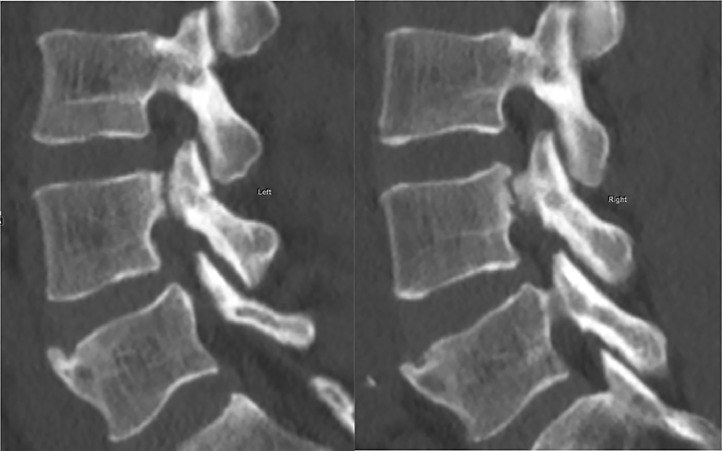
Bilateral para-midline sagittal images demonstrate the intact pars interarticularis on both sides at L4-5.

**Fig. 7:**
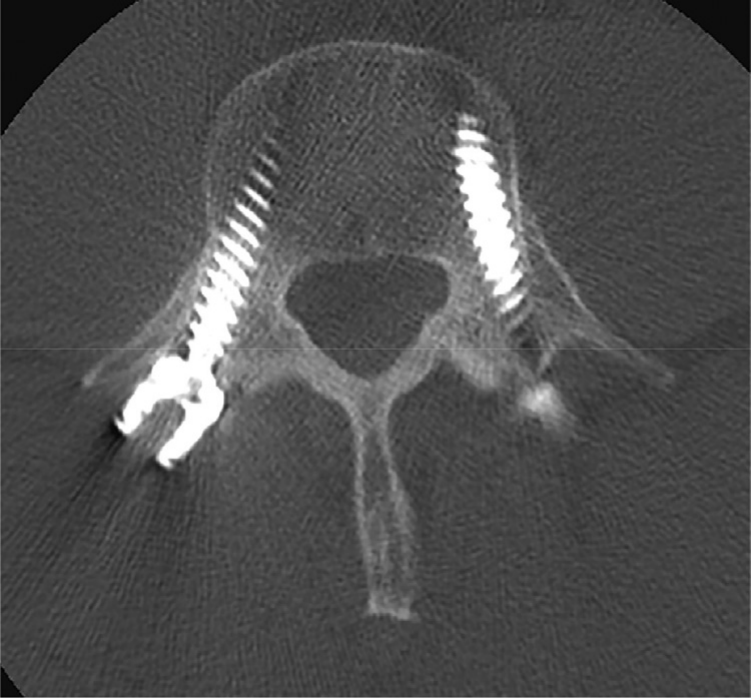
Axial postsurgical CT image showing bilateral transpedicular screws in place.

Fractures involving the pedicle are uncommon and can arise secondary to a contralateral fracture of the pars interarticularis (ie, spondylolysis) [1]. Bilateral pedicle fractures are rare and have been shown to occur in the context of underlying bony disease, trauma, or postspinal surgery [2,3]. Stress or fatigue fracture results from abnormal repetitive force on a normal bone [4]. Our case depicts bilateral pedicle fractures of the L4 vertebra in an otherwise healthy 41-year-old male. The subject was an amateur weightlifter but did not report any traumatic injury that could explain his backache (Fig. 1, Fig. 2, Fig. 3, Fig. 4, Fig. 5, Fig. 6, Fig. 7).

## Case report

Our case depicts a 41-year-old male presenting with a 3-month history of atraumatic low back pain with associated right-sided radiculopathy in the L3-L5 distribution. His neurological examination was otherwise normal. His background history includes depression, and his medications include an SSRI, paracetamol with codeine, and nonsteroidal anti-inflammatories to manage his pain. He was an ex-smoker and reported occasional cannabis use. His height was 181 cm and BMI 29.

Due to the persistent nature of back pain, he was booked for computed tomography of the lumbar spine. Computed tomography depicted bilateral pedicle fractures with sclerosed margins at L4. No focal bony lesion or osteopenia was noted. Magnetic resonance imaging showed the fractures with no significant bone marrow edema.

He was followed up by orthopedics and underwent surgical treatment with bilateral pedicle screws.

Given the unexpected findings and no history of trauma, further questioning revealed that the patient was an amateur weightlifter but did not report any sudden onset of symptoms during exercise.

## Discussion

This case depicts atraumatic bilateral pedicle fractures of the L4 vertebrae in an otherwise healthy 41-year-old male. Pedicle fractures are a rare phenomenon and have been reported in prior case reports. Previously established causes include trauma, degenerative spine disease, prolonged bisphosphonate use, and postspinal surgery. There are several reports of athletes – particularly ballet dancers – developing stress fractures of the lumbar pedicles [5,6]. Our patient self-reported amateur weightlifting but did not recall a specific traumatic injury as a consequence of this. Our only explanation for his presentation is a stress fracture secondary to weightlifting. Of note, prior reports have also demonstrated bilateral pedicle fractures in sedentary individuals without known risk factors [7].

The pedicles (from Latin pedīculus ``little foot'') are the bony prominences arising from the vertebral body that join the 2 laminae to form the posterior neural arch – a ring-like structure protecting the neural tissue within the spinal canal. The pedicles are the second weakest structure within the vertebrae after the pars interarticularis. In cadaveric experiments testing mechanical force on the vertebrae, most shear forces propagated obliquely through the pars interarticularis. Once a sufficient amount of static force or the summation of multiple repetitive forces reaches the threshold for bone failure, a fracture will occur [4]. Fracture of the pars (ie, spondylolysis) is the most common location of fracture in the vertebrae [8].

Unilateral spondylolysis causes instability of the neural arch resulting in a redistribution of forces. This can lead to contralateral sclerosis, hypertrophy, or fracture [1]. Pedicle fracture represents a condition similar to spondylolysis called ``pediculolysis''; this can occur unilaterally in the setting of contralateral spondylosis or bilaterally [9]. Bilateral fractures are exceedingly rare, especially in the absence of the previously mentioned risk factors. We suspect that our patient developed his fractures due to improper weightlifting technique, causing shear forces to propagate through his pedicles instead of the pars.

## Conclusion

Although unusual for a vertebral fracture to involve pedicles, cadaveric models and prior case reports have demonstrated its occurrence. Normally shear forces propagate obliquely through the pars but we suspect that certain movements may place sufficient force through the pedicle resulting in failure [1], [2], [3], [4], [5], [6], [7], [8], [9]. Our case represents bilateral stress fractures within the pedicles in an individual whose only risk factor was amateur weightlifting.

## Patient Consent Statement

Written informed consent was obtained from our patient. All identifying features were removed.
